# *Fusobacterium nucleatum* induces chemoresistance in colorectal cancer by inhibiting pyroptosis via the Hippo pathway

**DOI:** 10.1080/19490976.2024.2333790

**Published:** 2024-03-27

**Authors:** Ni Wang, Lu Zhang, Xiao-Xu Leng, Yi-Le Xie, Zi-Ran Kang, Li-Cong Zhao, Lin-Hong Song, Cheng-Bei Zhou, Jing-Yuan Fang

**Affiliations:** a Division of Gastroenterology and Hepatology; Shanghai Institute of Digestive Disease; NHC Key Laboratory of Digestive Diseases; State Key Laboratory for Oncogenes and Related Genes; Renji Hospital, School of Medicine, Shanghai Jiao Tong University, Shanghai, China; b Department of Gastroenterology; Shanghai Ninth People’s Hospital, School of Medicine, Shanghai Jiao Tong University, Shanghai, China

**Keywords:** Fusobacterium nucleatum, colorectal cancer, pyroptosis, chemoresistance, YAP, GSDME

## Abstract

Chemotherapy resistance is one of the main reasons for the poor prognosis of colorectal cancer (CRC). Moreover, dysbiosis of gut bacteria was found to be a specific environmental risk factor. In this study, enrichment of *F. nucleatum* was elucidated to be significantly associated with CRC recurrence after chemotherapy. Functional experiments showed that *F. nucleatum* could inhibit pyroptosis induced by chemotherapy drugs, thereby inducing chemoresistance. Furthermore, mechanistic investigation demonstrated that *F. nucleatum* could regulate the Hippo pathway and promote the expression of BCL2, thereby inhibiting the Caspase-3/GSDME pyroptosis-related pathway induced by chemotherapy drugs and mediating CRC cell chemoresistance. Taken together, these results validated the significant roles of *F. nucleatum* in CRC chemoresistance, which provided an innovative theoretical basis for the clinical diagnosis and therapy of CRC.

## Introduction

Colorectal cancer (CRC) is the second most common cancer and leading cause of cancer-related death worldwide.^1,2^ Patients with early CRC respond well to chemotherapy and have a high 5-year survival rate. However, the prognosis of advanced tumors, especially stage IV, remains poor because of the high rate of tumor recurrence and distant metastasis.^3^ 5-Fluorouracil (5-Fu) combined with oxaliplatin or irinotecan is a conventional standard treatment for patients with advanced CRC.^4^ Chemoresistance is one of the major causes of poor prognosis in CRC.^5^ Therefore, it is essential to explore the mechanism of CRC chemoresistance.

Genetic and environmental factors are important causes for the development of CRC. Among them, with the development of next-generation sequencing technology and new insights into the pathogenesis of CRC, the gut microbiota was found to be a specific environmental risk factor.^6^ In the genomic analysis of the CRC microbiome, *Fusobacterium nucleatum* (*F. nucleatum*) was found to be enriched in tumor tissues.^7^ Significantly, our previous research has shown that *F. nucleatum* may promote chemoresistance in patients with CRC by activating the autophagy pathway and inhibiting apoptosis,^8^ which proved the important role of *F. nucleatum* in CRC chemoresistance. However, some CRC cells still have drug resistance after reversing the inhibition of apoptosis by *F. nucleatum* by blocking autophagy. Therefore, we further investigated whether there are other models of cell death that are regulated by *F. nucleatum* and involved in chemoresistance in CRC.

Pyroptosis is a novel necrotizing and lytic pro-inflammatory programmed cell death^9^ that is also known as cellular inflammatory “necrosis”.^10,11^ In brief, pyroptosis is a process in which activated Caspases cleave Gasdermins (GSDMs) proteins, inducing the binding of the N-terminal domain of GSDMs to the phospholipid bilayer, forming pores in the cell membrane, inducing changes in cell osmotic pressure and the release of various cytoplasmic contents, eventually leading to cell swelling until bursting.^12^ There are six known human GSDM genes: GSDMA, GSDMB, GSDMC, GSDMD, GSDME/deafness, autosomal dominant 5 (DFNA5) and DFNB59. In 2017, it was reported that chemotherapeutic agents could cleave GSDME through Caspase-3 and thus induce pyroptosis,^13,14^ suggesting that the inhibition of chemotherapy-induced GSDME-related pyroptosis may be involved in chemotherapy resistance.

In this study, the enrichment of *F. nucleatum* was significantly correlated with CRC postchemotherapy recurrence. Furthermore, through a series of functional and mechanistic experiments, the results showed that *F. nucleatum* inhibited chemotherapy-induced pyroptosis in CRC cells by regulating the YAP/BCL2/Caspase-3/GSDME pathway, thereby inducing chemoresistance in CRC.

## Results

### F. nucleatum *abundance is related to CRC progression and prognosis*

**Figure 1.**
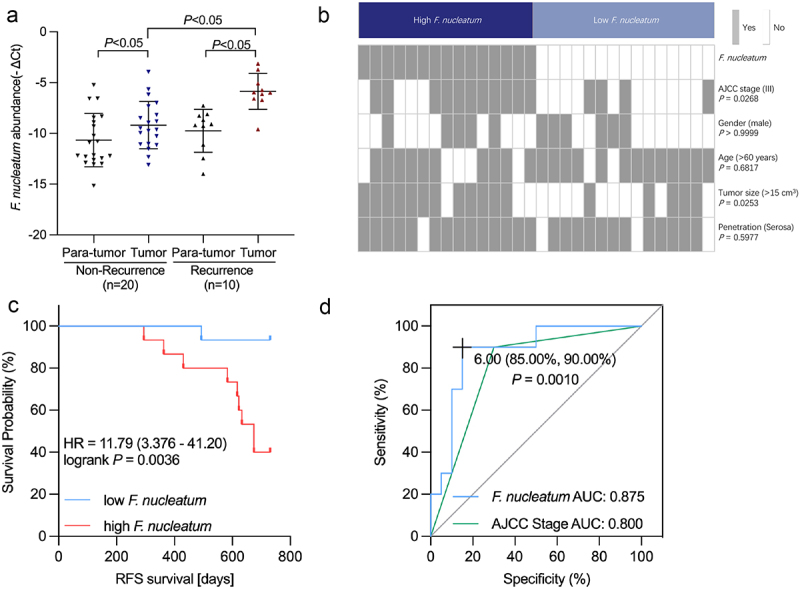
The abundance of *F. nucleatum* is associated with the progression and prognosis of CRC. (A) The abundance of *F. nucleatum* was measured in 30 pairs of CRC tissues. (B) Heatmap illustrating the association of various histopathological features and high-abundance and low-abundance *F. nucleatum* tumors. (C) Recurrence-free survival (RFS) was compared between groups with low and high *F. nucleatum* abundance. (D) Receiver operating characteristic (ROC) analysis was performed according to *F. nucleatum* abundance and AJCC in CRC.

To elucidate the correlation between *F. nucleatum* abundance and CRC clinical stage, we measured *F. nucleatum* levels in CRC samples of recurrent and non-recurrent patients. Consistent with the data in previous reports,^15^ qRT‒PCR analysis revealed the *F. nucleatum* enrichment in CRC tissues of recurrent patients compared with non-recurrent patients (Figure 1a). Moreover, in both the recurrent and non-recurrent groups, the abundance of *F. nucleatum* in CRC tissues increased compared to that in normal tissues (Figure 1A). The above data elucidated that *F. nucleatum* correlates with CRC recurrence. The association between *F. nucleatum* and clinicopathological features was further examined. In addition to AJCC, *F. nucleatum* abundance was positively correlated with tumor size as well (Figure 1b). Enrichment of *F. nucleatum* was associated with shorter recurrence-free survival (RFS) (Figure 1c). As CRC recurrence accounts for chemoresistance, we hypothesized that *F. nucleatum* may be involved in CRC chemoresistance. The predictive value of AJCC staging and *F. nucleatum* on CRC recurrence was analyzed using the receiver operating characteristic (ROC) curve. We found that the area under the curve (AUC) of the prediction model with *F. nucleatum* was higher than that with the AJCC stage (0.875 versus 0.800, *p* = .001) (Figure 1d). The above results suggested that *F. nucleatum* is related to CRC progression and prognosis.

### F. nucleatum inhibits chemotherapy-induced pyroptosis in CRC

To explore which possible models of death besides autophagy and apoptosis play an important role in the chemoresistance of CRC, we treated CRC cells with oxaliplatin with *F. nucleatum* intervention and found that *F. nucleatum* could affect chemotherapy-induced GSDME-related pyroptosis (Figures S1A). Wang *et al*. identified that GSDME could transform Caspase-3 mediated apoptosis induced by chemotherapy drugs into pyroptosis.^13^ Under the stimulation of chemotherapy, GSDME can be cleaved specifically by cleaved Caspase-3, resulting in the generation of membrane-penetrating GSDME-N fragments that induce pyroptosis.^13^ In CRC cells, lobaplatin has been shown to mediate pyroptosis through GSDME activation induced by Caspase-3.^16^ Therefore, we hypothesized that *F. nucleatum* could downregulate the GSDME-related pyroptosis induced by chemotherapy drugs to induce chemoresistance.

**Figure 2.**
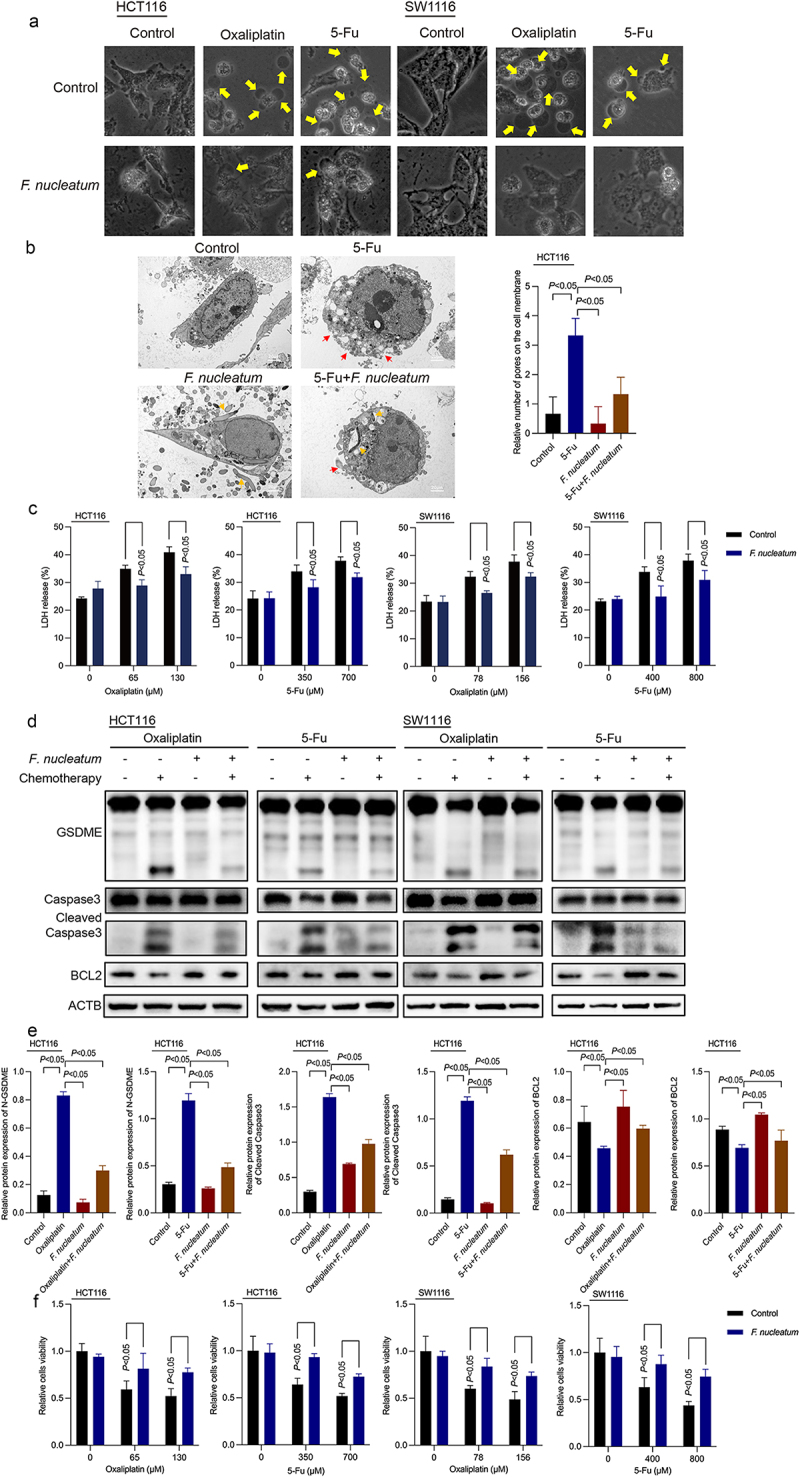
*F. nucleatum* represses chemotherapy-induced pyroptosis in CRC. (a) Representative bright-field images of CRC cells cocultured with chemotherapy drugs and *F. nucleatum*. Yellow arrows indicate large bubbles emerging from the cell membrane. (b) Transmission electron microscopy observation of CRC cells cocultured with 5-fu and *F. nucleatum*. Red and orange arrows indicate cell membrane pores and *F. nucleatum*, respectively. (c) The effects of chemotherapy drugs and *F. nucleatum* on LDH release from cells were detected. (d-e) the effect of *F. nucleatum* and chemotherapy on the expression of pyroptosis-related proteins in CRC cells was detected by Western blot. (f) Cell viability after treatment with chemotherapy and *F. nucleatum* in CRC cells was detected by CCK8 assay. All experiments were performed in biological triplicates.

To test the above hypothesis, CRC cells (HCT116 and SW1116) were treated with chemotherapy drugs (oxaliplatin and 5-Fu) and cocultured with *F. nucleatum in vitro*. Morphologically, we found that oxaliplatin and 5-Fu-treated CRC cells exhibited significant swelling with characteristic large bubbles on the cell membrane. *F. nucleatum* treatment inhibited chemotherapy-induced cell swelling (Figure 2a). Transmission electron microscopy (TEM) illustrated multiple pores formed on the membrane of chemotherapy drug-cultured CRC cells. While the number of pores on the cell membrane of HCT116 cells caused by chemotherapy drugs was significantly attenuated after coculture with *F. nucleatum* (Figure 2b). When pyroptosis occurs, pores formed in the cell membrane lead to the release of cell contents such as lactate dehydrogenase (LDH). We found that chemotherapeutic drugs could induce pyroptosis-induced LDH release, which could be inhibited by *F. nucleatum* coculture (Figure 2c). Moreover, we found that the elevated levels of Caspase-3 cleavage and GSDME cleavage induced by chemotherapy drugs were inhibited in *F. nucleatum*-treated CRC cells (Figures 2d, e, Figure S1B). These results indicated that *F. nucleatum* could inhibit the Caspase-3/GSDME pyroptosis pathway induced by oxaliplatin and 5-Fu. Furthermore, the results of the CCK8 assay showed that the cell viability of CRC cells infected with *F. nucleatum* was evidently higher than that of the control cells under chemotherapeutic agent coculture (Figure 2f), which indicated that *F. nucleatum* could induce the chemoresistance of CRC cells *in vitro*. Furthermore, using apoptosis agonists Grifolin,^17^ autophagy lysosomal inhibitor Chloroquine (CQ)^8^ and pyroptosis agonists Triclabendazole^18^ to block or induce apoptosis, autophagy and pyroptosis, we found that all these three pathways played a part in *F. nucleatum*-induced chemoresistance, among which pyroptosis played a more important role (Figure S1C).

### *BCL2 is involved in the inhibitory effect of* F. nucleatum *on chemotherapy-induced pyroptosis*

**Figure 3.**
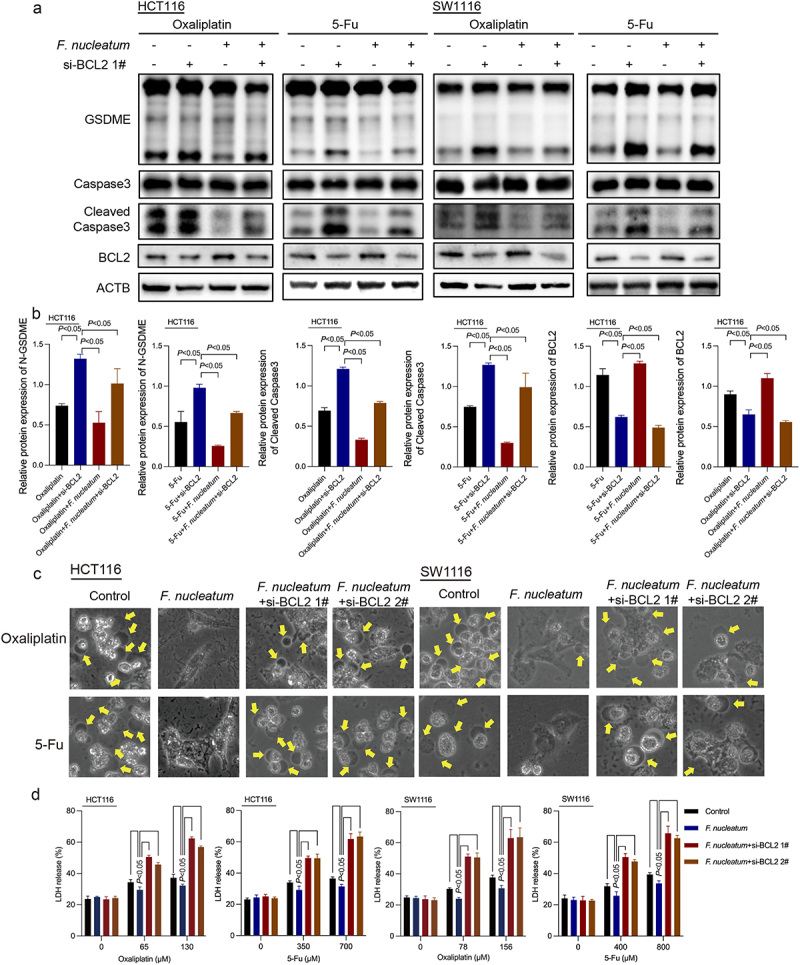
BCL2 knockdown affects the inhibitory effect of *F. nucleatum* on chemotherapy-induced pyroptosis. (a-b) the effect of *F. nucleatum* on the expression of pyroptosis-related proteins in chemotherapy-treated CRC cells after inhibition of BCL2 expression was detected by Western blotting. (c) The morphological changes in *F. nucleatum-*treated CRC cells after BCL2 inhibition were observed under a light microscope. (d) LDH release from chemotherapy-treated CRC cells after *F. nucleatum* intervention after BCL2 inhibition was detected. All experiments were performed in biological triplicates.

B-cell lymphoma-2 (BCL2) is an oncogene that can significantly inhibit cell apoptosis. Lobaplatin treatment could downregulate the expression of BCL2 in CRC.^16^ Triclabendazole can activate pyroptosis by modulating the level of the apoptotic protein BCL2, enhancing the cleavage of Caspase-3 and GSDME.^18^ Western blotting results revealed that *F. nucleatum* could ameliorate the reduction in BCL2 protein levels induced by chemotherapy drugs (Figures 2d, e, Figure S1B). Thus, we hypothesized that BCL2 may be related to the inhibition of chemotherapy-induced pyroptosis caused by *F. nucleatum* in CRC. To investigate whether BCL2 participates in the pyroptosis of CRC cells inhibited by *F. nucleatum*, siRNA-mediated knockdown was used to manipulate the expression of BCL2 exogenously (Figures S2A and S2B). Western blot results showed that knockdown of BCL2 remedied *F. nucleatum*-reduced Caspase-3 and GSDME activation in chemotherapy-treated CRC cells (Figures 3a & 3b, Figure S2C). Morphologically, the inhibitory effect of *F. nucleatum* on chemotherapy-induced cell swelling could be blocked by downregulating BCL2 (Figure 3c). Moreover, si-BCL2 transfection abolished the suppressive effect of *F. nucleatum* infection on LDH release (Figure 3d). These results suggested that *F. nucleatum* could inhibit chemotherapy-induced pyroptosis in CRC cells by promoting BCL2 expression. As reported in our previous literature,^8^ *F. nucleatum* could inhibit the apoptosis of CRC cells induced by chemotherapy (Figure S2D). Furthermore, knockdown of BCL2 could affect the regulation of *F. nucleatum* on apoptosis (Figure S2E).

### F. nucleatum induces chemoresistance by upregulating BCL2

**Figure 4.**
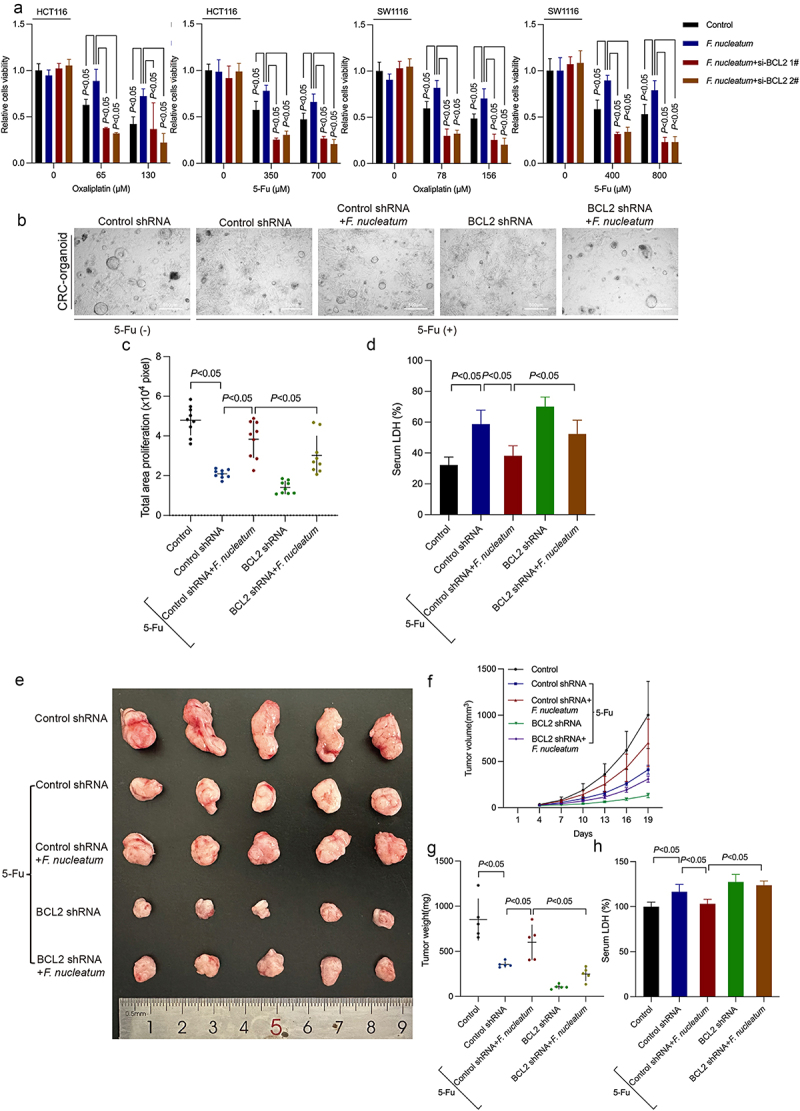
*F. nucleatum* induces chemotherapy resistance by regulating BCL2. (a) Survival of chemotherapy-treated CRC cells after *F. nucleatum* intervention and BCL2 inhibition was determined by CCK8 assay. (b) Representative images of CRC patient-derived organoids with *F. nucleatum* or BCL2 shRNA. (c) The size of proliferation on CRC patient-derived organoids was measured. (d) LDH release from CRC patient-derived organoids with *F. nucleatum* after BCL2 downregulation was detected. (e) Xenograft tumors in the nude mouse model were used to examine the effect of BCL2 inhibition on the chemoresistance of *F. nucleatum*-treated CRC cells. (f) Tumor volumes were calculated after injection every 3 days. (g) Tumor weights are represented as the means of tumor weights ± S.D. (h) The release of serum LDH in nude mice was measured. Bars indicate S.D.

We further explored whether *F. nucleatum* could induce chemoresistance in CRC cells by inhibiting pyroptosis through modulating BCL2. We cocultured *F. nucleatum* with BCL2-siRNA-transfected cells and detected cell viability by CCK8, which showed that knockdown of BCL2 could inhibit *F. nucleatum* induced chemoresistance (Figure 4a). We confirmed this chemoresistance-inducing property of *F. nucleatum* in patient tissue-derived CRC organoids (Figures 4b, c). Downregulated BCL2 could suppress *F. nucleatum* induced chemoresistance (Figures 4b, c) and block the inhibition of LDH release by *F. nucleatum* in patient tissue-derived CRC organoids as well (Figure 4d). Then, we further established CRC xenograft models to identify the effect of *F. nucleatum* and BCL2 on the chemoresistance of CRC *in vivo*. HCT116 cells stably expressing sh-BCL2 or control were inoculated subcutaneously, followed by treatment with 5-Fu and *F. nucleatum*. Nineteen days following the injection, 5-FU treatment significantly inhibited tumor growth, and this inhibitory effect was blocked by infection with *F. nucleatum in vivo* (Figures 4e, f). Consistent results were also obtained when we measured tumor weight at the end of the assays (Figure 4g). Moreover, *F. nucleatum* can inhibit the release of LDH induced by chemotherapeutics. The inhibitory effect of *F. nucleatum* on chemotherapy-induced LDH release can be reversed by downregulating BCL2 (Figures 4h). These data illustrated that *F. nucleatum* was related to the resistance of CRC to 5-FU treatment. BCL2 knockdown reversed *F. nucleatum*-stimulated CRC chemoresistance in a 5-FU-treated xenograft mouse model (Figures 4e–h). *F. nucleatum*-induced CRC chemoresistance could be rescued by BCL2 inhibitor Venetoclax in 5-FU-treated *Apc*^*Min/+*^ mice (Figure S3A-C). The repression of *F. nucleatum* on 5-FU-induced LDH release can be reversed by BCL2 inhibitor Venetoclax as well (Figure S3D). All of the above results suggested that *F. nucleatum* could suppress chemotherapy drug-induced pyroptosis by regulating BCL2, thereby inducing CRC chemoresistance.

### F. nucleatum inhibits chemotherapy-induced pyroptosis by modulating the Hippo pathway

It has been reported that *F. nucleatum* intervention can activate the Hippo pathway in CRC cells.^19^ Moreover, Yes-associated protein (YAP) can act as a cotranscription factor in the BCL2 promoter region to promote the transcription of BCL2 in CRC cells.^20,21^ Inhibition of the YAP pathway was shown to promote the cleavage of Caspase-3.^22^ Therefore, we hypothesized that *F. nucleatum* may regulate the downstream BCL2-induced pyroptosis pathway by activating the YAP pathway.

**Figure 5.**
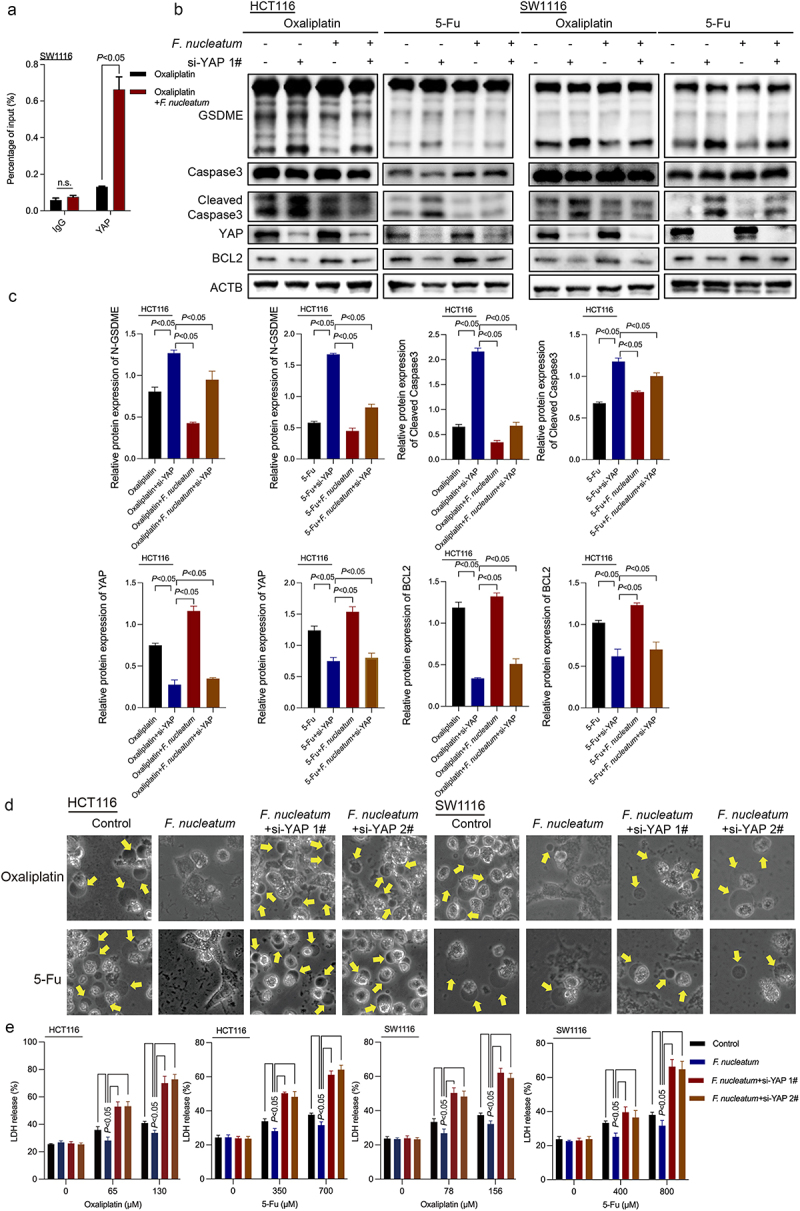
*F. nucleatum* affects BCL2-mediated pyroptosis by regulating YAP. (a) ChIP assay to detect the enrichment of YAP in the BCL2 promoter region in chemotherapeutic drug-treated CRC cells after *F. nucleatum* intervention. (b-c) CRC cells were transfected with si-YAP. After coculturing with *F. nucleatum* and chemotherapy drugs, pyroptosis target proteins were detected by western blotting. (d) Representative bright-field images were observed under a light microscope. Yellow arrows indicate large bubbles emerging from the cell membrane. (e) The effect of YAP knockdown on LDH release in CRC cells infected with *F. nucleatum* was detected. All experiments were performed in biological triplicates.

Given that YAP modulates target gene transcription through promoter binding, we performed ChIP‒qPCR to explore whether *F. nucleatum* could regulate BCL2 expression by activating the Hippo pathway. The results demonstrated that under a certain concentration of oxaliplatin, treatment with *F. nucleatum* indeed promoted the enrichment of YAP in the BCL2 promoter region (Figure 5a). Consistently, downregulation of YAP significantly inhibited *F. nucleatum*-stimulated BCL2 protein expression (Figures 5b & 5c). These data indicated that *F. nucleatum* could activate downstream transcriptional expression of BCL2 by promoting YAP binding to the promoter region of BCL2. We further knocked down YAP in HCT116 and SW1116 cell lines with siRNA (Figures S4A and S4B). Western blot results further showed that suppression of YAP alleviated the inhibition of Caspase-3 and GSDME cleavage induced by *F. nucleatum* (Figures 5b & 5c, Figure S4C). The morphological images and LDH release assays showed that YAP knockdown repressed the inhibitory effect of *F. nucleatum* on pyroptosis as well (Figure 5d, e). These findings implied that *F. nucleatum* could inhibit chemotherapy-induced pyroptosis in CRC cells by modulating the YAP/BCL2/Caspase-3/GSDME pathway.

### F. nucleatum regulates the Hippo pathway to induce chemoresistance

**Figure 6.**
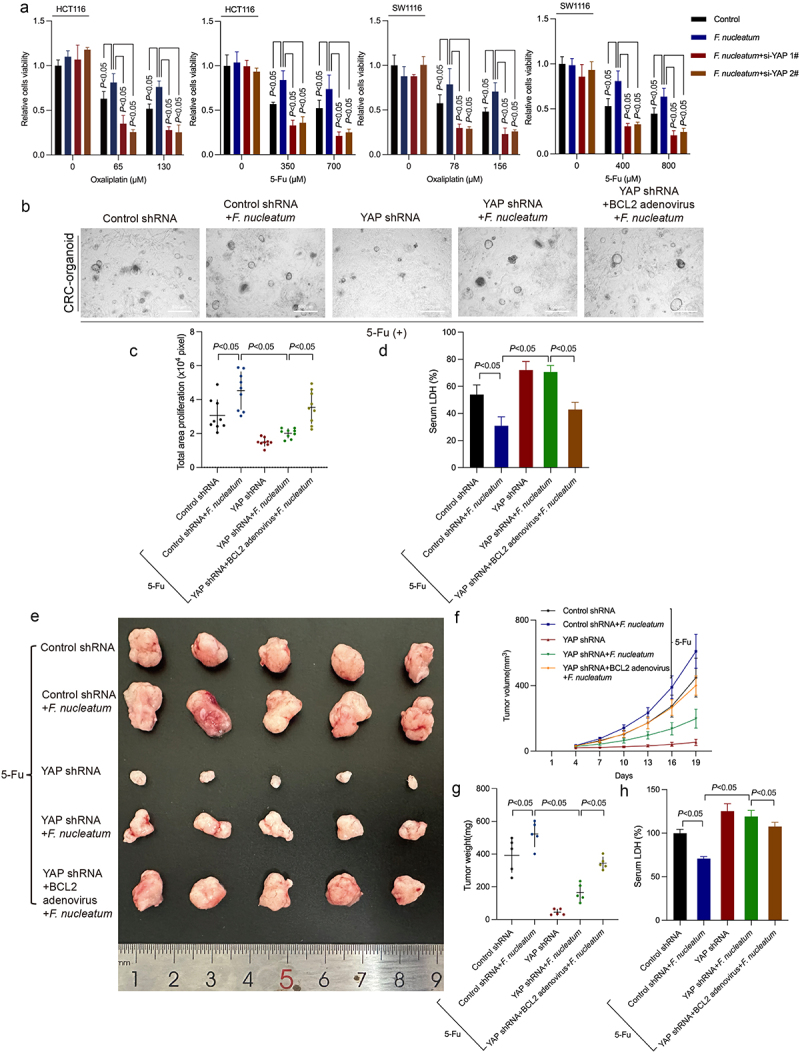
YAP mediates *F. nucleatum*-induced chemoresistance. (a) CCK8 assay was used to detect the effect of si-YAP on the viability of CRC cells treated with chemotherapy drugs after *F. nucleatum* intervention. (b) Representative images of chemotherapy-treated CRC patient-derived organoids with *F. nucleatum*, YAP shRNA or BCL2 adenovirus. (c) The size of proliferation on chemotherapy-treated CRC cells CRC patient-derived organoids was measured. (d) LDH release from CRC patient-derived organoids with *F. nucleatum* after YAP downregulation or BCL2 upregulation was detected. (e) Representative data of xenograft tumors in the nude mouse model bearing HCT116 cells in different groups. (f-g) statistical analysis of mouse tumor volumes (f) and weights (g). (h) The release of LDH in the serum of nude mice was detected. Bars indicate S.D.

To explore the role of YAP in the chemoresistance induced by *F. nucleatum*, we tested the effect of YAP downregulation on the viability of *F. nucleatum-* infected CRC cells after chemotherapeutic drug coculture. The CCK8 assay showed that downregulation of YAP inhibited *F. nucleatum* infection-induced chemoresistance of CRC cells *in vitro* (Figure 6a). In CRC organoids, downregulation of YAP could inhibit the chemoresistance induced by *F. nucleatum* and rescue the inhibition of LDH release by *F. nucleatum* (Figure 6b–d). Upregulation of BCL2 prevents downregulation of YAP-mediated rescue of *F. nucleatum-*induced chemoresistance and *F. nucleatum*-suppressed LDH release (Figure 6b–d). *In vivo* assays ascertained that knockdown of YAP rescued *F. nucleatum*-induced chemoresistance in 5-FU-treated mice (Figure 6e–g). Furthermore, BCL2 adenovirus blocked the downregulated YAP-mediated reversion of *F. nucleatum*-induced chemoresistance in tumor-bearing mouse models treated with 5-Fu (Figure 6e–g). Moreover, the inhibition of chemotherapy-induced LDH release by *F. nucleatum* was reversed by YAP knockdown (Figure 6h). BCL2 overexpression blocked the YAP knockdown-mediated reversal of *F. nucleatum*-inhibited LDH release (Figure 6h). In 5-FU-treated *Apc*^*Min/+*^ mice, upregulated BCL2 could rescue YAP inhibitor Verteporfin-mediated reversal of *F. nucleatum*-induced chemoresistance and *F. nucleatum*-inhibited LDH release as well (Figure S5A-D). These data suggested that *F. nucleatum* could inhibit chemotherapy-induced Caspase-3/GSDME-related pyroptosis by regulating the Hippo pathway and promoting BCL2 expression, thereby potentiating chemoresistance. Furthermore, we obtained stable GSDME-knockout cells by CRISPR/Cas9, which was verified via WB (Figures S6A and S6B). Interestingly, the results showed that GSDME knockout only partially blocked *F. nucleatum*-induced chemotherapy resistance, implying that *F. nucleatum* may induce chemotherapy resistance through autophagy or apoptosis pathways as well (Figure S6C).

### *The abundance of* F. nucleatum, *YAP, and BCL2 is clinically correlated*

**Figure 7.**
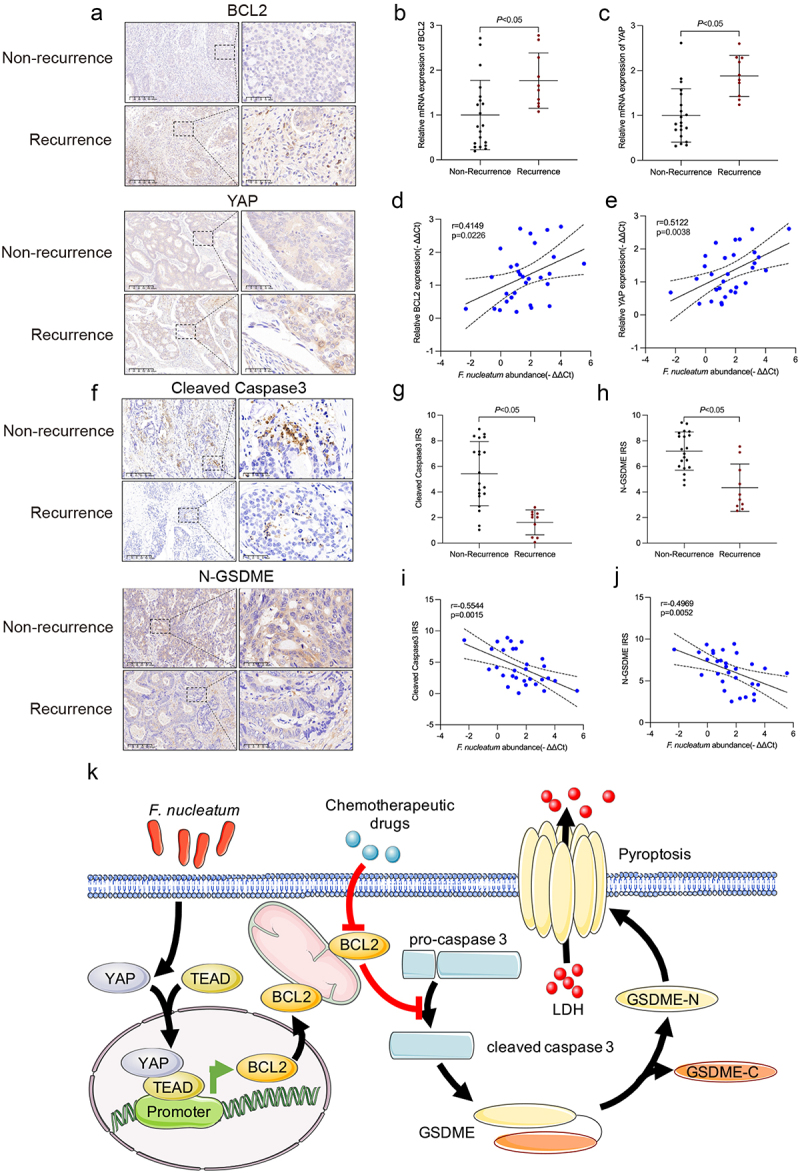
*F. nucleatum*, YAP, BCL2, caspase-3 and GSDME were clinically relevant. (a) Protein levels of YAP and BCL2 in CRC tissues from non-recurrent and recurrent patients were detected by immunohistochemistry. (b and c) the mRNA expression of YAP and BCL2 was statistically analyzed by qRT‒PCR. (d and e) correlation of the expression levels of *F. nucleatum*, YAP and BCL2 in human CRC tissues. (f) Protein levels of cleaved caspase-3 and N-GSDME in CRC tissues from non-recurrent and recurrent patients were detected by immunohistochemistry. (g and h) statistical analysis of immunohistochemical immunoreactive score of Remmele and Stegner (IRS) scores of cleaved caspase-3 and N-GSDME proteins. (i and j) correlation of the expression levels of *F. nucleatum*, cleaved caspase-3 and N-GSDME in human CRC tissues. (k) The schematic model shows that *F. nucleatum* may affect chemoresistance in CRC cells by inhibiting chemotherapy-induced pyroptosis.

To investigate the clinical relevance of *F. nucleatum*, YAP and BCL2 in CRC, we examined their levels in CRC tissues and adjacent normal colorectal tissues by immunohistochemistry and PCR. The immunohistochemistry and qRT‒PCR results illustrated that the mRNA and protein levels of BCL2 and YAP were highly expressed in recurrent patients compared with non-recurrent patients (Figures 7a–c). Moreover, combining the PCR results with *F. nucleatum* abundance in the clinical cohort, the amount of *F. nucleatum* was positively associated with the expression of YAP and BCL2 in CRC tissues (Figures 7d & 7e). The immunohistochemistry results showed that downregulated protein levels of Cleaved Caspase-3 and N-GSDME were in recurrent patients compared with non-recurrent patients (Figures 7f–h). Moreover, the amount of *F. nucleatum* was negatively correlated with Cleaved Caspase-3 and N-GSDME in CRC (Figures 7i & 7j). The above data supported that *F. nucleatum* could regulate the chemotherapy-induced BCL2/Caspase-3/GSDME pyroptosis pathway by activating the Hippo pathway, thus mediating chemoresistance in CRC (Figure 7k).

## Discussion

The intestinal microbiota was reported to correlate with chemotherapy resistance in CRC.^23^ For example, *Caenorhabditis elegans* can influence the host response to 5-Fu.^24^ The enrichment of *F. nucleatum* was shown to be involved in drug resistance to standard 5-Fu adjuvant chemotherapy in patients with CRC after radical resection.^25^ In this study, using a clinical cohort of 30 patients, the results illustrated that *F. nucleatum* enrichment was related to the recurrence of CRC after chemotherapy. As chemoresistance is a major cause of CRC recurrence, we hypothesized that enrichment of *F. nucleatum* may be linked to CRC chemotherapy resistance.

Based on our previous study, after reversing the inhibition of apoptosis by *F. nucleatum* by blocking autophagy, some CRC cells remained resistant to chemotherapy.^8^ Therefore, we further explored whether other cell death models besides autophagy and apoptosis were regulated by *F. nucleatum* and involved in chemoresistance in CRC. In 2017, Shao *et al*. demonstrated that chemotherapy can cleave GSDME through the cleavage of activated Caspase-3, producing GSDME-N fragments in cancer cells to induce pyroptosis.^13^ In CRC, the chemotherapeutic drug lobaplatin and small molecule protein Apoptin could induce GSDME-mediated pyroptosis in CRC cells.^16,26^ These studies suggested that inhibition of pyroptosis-related gene GSDME splicing in tumors may trigger chemoresistance in tumors. Therefore, we hypothesized that *F. nucleatum* could induce chemotherapeutic drug resistance by affecting Caspase-3/GSDME-mediated pyroptosis. Our results confirmed that *F. nucleatum* infection inhibited chemotherapeutic drug-induced cell swelling and LDH release and suppressed Caspase-3 and GSDME cleavage at the protein level. These results suggested that *F. nucleatum* could inhibit Caspase-3/GSDME pathway-mediated pyroptosis induced by chemotherapy drugs. These findings explained the correlation between pyroptosis and chemotherapy resistance from the perspective of gut microbiota. Based on our previous findings that *F. nucleatum* could induce CRC chemoresistance by inducing autophagy, we further supplemented the mechanism between *F. nucleatum* and chemoresistance.

BCL2 is widely recognized as an important inhibitor of apoptosis that maintains mitochondrial membrane integrity. Increased expression levels of BCL2 have been associated with resistance to several anticancer drugs.^27–29^ Our results illustrated that BCL2 could mediate the *F. nucleatum*-repressed chemotherapy-induced Caspase-3/GSDME pyroptosis pathway and affect *F. nucleatum-*induced chemoresistance, which may provide a theoretical basis for the clinical application of BCL2 inhibitors.

The oncogenic transcriptional coactivator YAP is nuclear effector of the Hippo signaling pathway, which could play a key role in tissue homeostasis and tumorigenesis.^30,31^ Aberrantly expressed YAP has been characterized in a variety of cancers.^31^ YAP overexpression is also linked to poor prognosis in CRC.^32^ Therefore, a better understanding of the Hippo pathway signaling mechanism may identify new therapeutic targets for the treatment of cancer. This study revealed that *F. nucleatum* infection could promote YAP nuclear translocation and act as a cotranscription factor in the BCL2 promoter region to promote the transcription of BCL2 in CRC cells. YAP knockdown downregulated BCL2 expression to promote Caspase-3 cleavage and GSDME cleavage and rescued the inhibition of chemotherapy-induced pyroptosis and chemoresistance of *F. nucleatum*, which may be used as a potential molecular target for CRC treatment. However, how *F. nucleatum* modulates the Hippo pathway and the specific mechanism is still unknown. Previous research may give us some suggestions. Fusobacterial LPS has been illustrated to interplay with cell membrane receptor TLR4, thus activating the NF-κB signaling pathway.^33–35^ It has been reported that there is a reciprocal regulation between Hippo-YAP/TAZ and NF-κB signaling pathway.^36,37^ Therefore, we hypothesized that Fusobacterial LPS may interact with cell membrane receptor TLR4 to trigger NF-κB, thus regulating the Hippo pathway and pyroptosis, which needs to be further confirmed. Moreover, other possible interactions and mechanisms between *F. nucleatum* and CRC cells need to be further explored as well.

Taken together, *F. nucleatum* abundance was found to be correlated with postoperative chemotherapy resistance in patients with CRC. *F. nucleatum* could regulate the Hippo pathway and promote the expression of BCL2, thereby inhibiting the chemotherapy-induced Caspase-3/GSDME pyroptosis-related pathway and mediating the chemoresistance of CRC cells. Our data indicated the potential utilities of *F. nucleatum* as a prognostic biomarker and a novel therapeutic target in CRC. The important role of *F. nucleatum*-regulated chemotherapy-induced pyroptosis in colorectal chemoresistance may provide a new theoretical basis for the clinical prevention and treatment of CRC.

## Material and methods

### Tissue gathering and ethics statement

Thirty CRC patients who underwent surgery (2021–2023) at Shanghai Renji Hospital were included. Tissue samples were rapidly stored in liquid nitrogen until RNA was extracted. This study was approved by the ethics committee of Renji Hospital, School of Medicine, Shanghai Jiao Tong University. And the procedures were in accordance with the Helsinki Declaration of 1975. Informed consent was obtained from participating patients.

### *Detection of* F. nucleatum *abundance*

A QIAamp DNA FFPE Tissue Kit (QIAGEN, Hilden, Germany 56,404) was used for extraction of total DNA from FFPE CRC specimens. Quantitative real-time PCR (qPCR) was used to detect *F. nucleatum* abundance (see Supplementary Table S1 for primers).

### RNA extraction and qRT‒PCR analysis

RNA was isolated from specimens or cultured cells with TRIzol reagent (Invitrogen, USA 15,596,026). Then, RNA was reverse transcribed to cDNA a Reverse Transcription Kit (Takara, Japan, RR037A). For real-time PCR analysis, we used TB Green (Takara, Japan, RR820A). The expression data were normalized to the expression of glyceraldehyde-3-phosphate dehydrogenase (GAPDH). The primer sequences are listed in Supplementary Table S1.

### Bacterial strains and the growth conditions

*F. nucleatum* (ATCC 25,586) were purchased from the American Type Culture Collection (ATCC, Manassas, Virginia) and cultured under anaerobic conditions, consisting of 90% N_2_, 5% CO_2_, and 5% H_2_ at 37°C, as previously described.^8^

### Cell culture

CRC cell lines HCT116 and SW1116 were purchased from ATCC. HCT116 and SW1116 cells were cultured in RPMI-1640 medium and DMEM, respectively, with 10% FBS in 5% CO_2_. All experiments were performed with mycoplasma-free cells. Bacterial treatment conditions for all *F. nucleatum* treatment experiments were treated in simple medium with a multiplicity of infection (MOI) of 200 for 2 h.

Oxaliplatin (S1224), 5-FU (S1209), Grifolin (HY-N9271), Chloroquine (CQ) (HY-17589A), and Triclabendazole (HY-B0621) were obtained from MedChemExpress (Shanghai, China). Si-NC, BCL2 siRNAs and YAP siRNA were constructed by GenePharma (Shanghai, China). The sh-BCL2, sh-YAP and BCL2 adenoviruses were obtained from Obio Technology Company (Shanghai, China). DharmaFECT 1 siRNA transfection reagent (Invitrogen, Carlsbad, CA, *T*-2001-04) and X-tremeGENE HP DNA Transfection Reagent (Roche, Basel, Switzerland 6,366,236,001) were used for siRNA and plasmid transfection, respectively. The siRNA and shRNA sequences are shown in Supplementary Table S1.

### Collection and culture of CRC patient-derived organoid

Organoids derived from patients’ CRC tissues were isolated and cultured as previously described.^38,39^ Organoids were embedded into Matrigel (Corning, New York, USA 354,234). The medium was renewed every 2 days. After 5 days of culture, Matrigel was disrupted by mechanical force or digestion buffer to expose the organoids. Organoids were then collected by centrifugation and re-cultured for further experiments.

### Microscopy imaging

To observe the morphology of pyroptosis, we seeded the cells on 6-well plates and took bright field pictures. Furthermore, pore formation of pyroptosis was observed under electron microscopy.

### LDH release assay

LDH levels were measured by a CytoTox96 LDH-release kit (Promega, Madison, WI, USA, G1780). The absorbance value at 450 nm was detected. Each assay was repeated three times.

### Cell proliferation analysis

Cell viability was measured by a CCK8 kit (Cell Counting Kit-8, Dojindo, Japan, HY-000868). Assays were repeated independently three times.

### Western blot assay and antibodies

The protein was electrophoretically separated using SDS‒PAGE, transferred to a PVDF membrane (Bio-Rad, USA 1,620,177) and incubated with specific antibodies. Signals were measured by an ECL Kit (Pierce Biotech, Rockford, Illinois, USA 32,209). The anti-ACTB antibody (CST, USA, 5125S) was employed as the control. Antibodies were purchased from two manufacturers, Cell Signaling Technology: Cleaved-Caspase-3 (9664), YAP (14074), BCL2 (15071), and Abcam: GSDME (ab215191).

### Flow cytometric analysis

After transfection with si-NC or si-BCL2, CRC cells treated with chemotherapy drug or *F. nucleatum* were harvested by trypsinization. After double staining with fluorescein isothiocyanate (FITC)-Annexin V and propidium iodide (BD Biosciences, Franklin Lakes, NJ, USA, BD556547), the cells were analyzed by flow cytometry (FACScan; BD Biosciences).

### In vivo *tumor formation assays*

In order to reduce the uncertainties in the experiment and maintain the consistency and reliability of the results, male nude mice were selected in this study. Four-week-old male nude mice purchased from the Experimental Animal Center of Shanghai Institute for Biological Sciences (Shanghai, China) were maintained under SPF conditions. HCT116 cells transfected with sh-BCL2 or sh-YAP were resuspended at 5 × 10^6^ cells/mL and subcutaneously inoculated into the mouse armpit. Intratumoral injection of adenovirus, peritumoral injection of *F. nucleatum*, and intraperitoneal injection of chemotherapy drugs were performed twice a week. Then, the tumor volumes were calculated as 0.5 × D × d^2^ (V, volume; D, longitudinal diameter; and d, latitudinal diameter) every 3 days. On day 19 postinjection, tumor weights were detected after mouse sacrifice. *Apc*^*Min/+*^ mice at 5–6 weeks old were treated with oral gavage of BCL2 inhibitor Venetoclax (MedChemExpress, China, HY-15531), intraperitoneal injections of YAP inhibitor Verteporfin (MedChemExpress, China, HY-B0146), enema of BCL2 adenovirus or oral gavage of *F. nucleatum*. *F. nucleatum* solution was collected and resuspended in PBS and then gavage to mice (1 × 10^8^ colony-forming unit (CFU)/100 μL PBS per mouse). After 12 weeks for the development of neoplastic lesions, tumor number and size were detected after mouse sacrifice. The study was implemented in strict accordance with Guide Recommendations for the National Institute of Health Guidelines for the Care and Use of Laboratory Animals. The protocol was approved by the Animal Care and Use Committee of Renji Hospital, School of Medicine, Shanghai Jiao Tong University.

### Chromatin immunoprecipitation assays (ChIP)

ChIP was implemented using EZ-ChIP KIT (17–371, Millipore, USA). YAP antibodies were purchased from Cell Signaling Technology. Quantification of chromatin DNA was detected by qPCR. The primer sequence sequences are shown in Supplementary Table S1.

### CRISPR/Cas9-mediated accomplishment of GSDME-knockout cells

The CRISPR/Cas9 genomic editing system was employed to create GSMDE knockout HCT116 cells, utilizing the single-guide RNA (sgRNA) (TGAGTACATCGCCAAGGGTG) targeting the deletion of GSDME gene, which was synthesized by Obio Technology Corporation (Shanghai, China). The constructed vector was pLenti-U6- spgRNA (GSDME)-CMV-Puro-P2A-3Flag-spCas9-wpre. A non-targeting sgRNA (sgCon) was the control. Stably transfected cells were filtered by 1 μg/ml puromycin after 72 h. Then monoclonal cells were selected through limiting dilution. Western blot was used to verify the knockout of GSDME in HCT116 cells.

### Immunohistochemistry

All tissue samples were embedded in the paraffin and sliced into 5-μm sections. The sections were blocked with QuickBlock blocking buffer (P0260, Beyotime, China) for 15 min at RT for subsequent immunostaining, followed by incubation with primary antibodies against YAP (1:200, CST, USA 14,074), BCL2 (1:400, CST, USA 15,071), Cleaved-Caspase-3 (1:200, CST, USA, 9664), N-GSDME (1:200, Abcam, USA, ab222408) at 4°C overnight. Then the MaxvisionTM2 HRP-Polymer anti-Mouse/Rabbit IHC Kit (KIT-5920, Maixin, China) was used to perform DAB staining and the sections were counterstained with hematoxylin.

### Statistical analysis

T test or one-way ANOVA test was used to assess the significance of the differences between groups. All outcome data are expressed as the mean ± standard deviation (mean ± SD). Two-sided *p* values were calculated, with a value of less than 0.05 considered to indicate statistical significance.

## Abbreviations

AUC: area under the curve
CCK8: Cell Counting Kit-8
ChIP: Chromatin immunoprecipitation assays
DFNA5: deafness, autosomal dominant 5
GAPDH: glyceraldehyde-3-phosphate dehydrogenase
GSDM: Gasdermin
LDH: lactate dehydrogenase
MOI: multiplicity of infection
RFS: recurrence-free survival
ROC: receiver operating characteristic
TEM: Transmission electron microscopy

## Supplementary Material

Supplementary Figure S2.tif

Supplementary table S1 The list of primers and siRNA sequence.xlsx

Supplementary Figure Legends clean.docx

Supplementary Figure S5.tif

Supplementary Figure S1.tif

Supplementary Figure S3.tif

Supplementary Figure S4.tif

Supplementary Figure S6.tif

## Data Availability

Data supporting these findings are available from the corresponding author upon reasonable request.

## References

[1] Sung H, Ferlay J, Siegel RL, Laversanne M, Soerjomataram I, Jemal A, Bray F. Global cancer statistics 2020: GLOBOCAN estimates of incidence and mortality worldwide for 36 cancers in 185 countries. CA Cancer J Clin. 2021;71(3):209–18. doi:10.3322/caac.21660. 33538338

[2] Siegel RL, Miller KD, Fuchs HE, Jemal A. Cancer statistics, 2022. CA Cancer J Clin. 2022;72(1):7–33. doi:10.3322/caac.21708. 35020204

[3] Brody H. Colorectal cancer. Nature. 2015;521(7551):S1. doi:10.1038/521S1a. 25970450

[4] Andre T, Boni C, Navarro M, Tabernero J, Hickish T, Topham C, Bonetti A, Clingan P, Bridgewater J, Rivera F. et al. Improved overall survival with oxaliplatin, fluorouracil, and leucovorin as adjuvant treatment in stage II or III colon cancer in the MOSAIC trial. J Clin Oncol. 2009;27(19):3109–3116. doi:10.1200/JCO.2008.20.6771. 19451431

[5] Meng N, Chen M, Chen D, Chen X-H, Wang J-Z, Zhu S, He Y-T, Zhang X-L, Lu R-X, Yan G-R. et al. Small protein hidden in lncRNA LOC90024 promotes “cancerous” RNA splicing and tumorigenesis. Adv Sci (Weinh). 2020;7(10):1903233. doi:10.1002/advs.201903233. 32440474 PMC7237858

[6] Coker OO, Nakatsu G, Dai RZ, Wu WKK, Wong SH, Ng SC, Chan FKL, Sung JJY, Yu J. Enteric fungal microbiota dysbiosis and ecological alterations in colorectal cancer. Gut. 2019;68(4):654–662. doi:10.1136/gutjnl-2018-317178. 30472682 PMC6580778

[7] Kostic AD, Gevers D, Pedamallu CS, Michaud M, Duke F, Earl AM, Ojesina AI, Jung J, Bass AJ, Tabernero J. et al. Genomic analysis identifies association of Fusobacterium with colorectal carcinoma. Genome Res. 2012;22(2):292–298. doi:10.1101/gr.126573.111. 22009990 PMC3266036

[8] Yu T, Guo F, Yu Y, Sun T, Ma D, Han J, Qian Y, Kryczek I, Sun D, Nagarsheth N. et al. Fusobacterium nucleatum promotes chemoresistance to colorectal cancer by modulating autophagy. Cell. 2017;170(3):548–563.e16. doi:10.1016/j.cell.2017.07.008. 28753429 PMC5767127

[9] Cookson BT, Brennan MA. Pro-inflammatory programmed cell death. Trends Microbiol. 2001;9(3):113–114. doi:10.1016/S0966-842X(00)01936-3. 11303500

[10] Chen X, He WT, Hu L, Li J, Fang Y, Wang X, Xu X, Wang Z, Huang K, Han J. et al. Pyroptosis is driven by non-selective gasdermin-D pore and its morphology is different from MLKL channel-mediated necroptosis. Cell Res. 2016;26(9):1007–1020. doi:10.1038/cr.2016.100. 27573174 PMC5034106

[11] Bergsbaken T, Fink SL, Cookson BT. Pyroptosis: host cell death and inflammation. Nat Rev Microbiol. 2009;7(2):99–109. doi:10.1038/nrmicro2070. 19148178 PMC2910423

[12] Broz P, Dixit VM. Inflammasomes: mechanism of assembly, regulation and signalling. Nat Rev Immunol. 2016;16(7):407–420. doi:10.1038/nri.2016.58. 27291964

[13] Wang Y, Gao W, Shi X, Ding J, Liu W, He H, Wang K, Shao F. Chemotherapy drugs induce pyroptosis through caspase-3 cleavage of a gasdermin. Nature. 2017;547(7661):99–103. doi:10.1038/nature22393. 28459430

[14] Rogers C, Fernandes-Alnemri T, Mayes L, Alnemri D, Cingolani G, Alnemri ES. Cleavage of DFNA5 by caspase-3 during apoptosis mediates progression to secondary necrotic/pyroptotic cell death. Nat Commun. 2017;8(1):14128. doi:10.1038/ncomms14128. 28045099 PMC5216131

[15] Yamamoto S, Kinugasa H, Hirai M, Terasawa H, Yasutomi E, Oka S, Ohmori M, Yamasaki Y, Inokuchi T, Harada K. et al. Heterogeneous distribution of Fusobacterium nucleatum in the progression of colorectal cancer. J Gastroenterol Hepatol. 2021;36(7):1869–1876. doi:10.1111/jgh.15361. 33242360

[16] Yu J, Li S, Qi J, Chen Z, Wu Y, Guo J, Wang K, Sun X, Zheng J. Cleavage of GSDME by caspase-3 determines lobaplatin-induced pyroptosis in colon cancer cells. Cell Death Disease. 2019;10(3):193. doi:10.1038/s41419-019-1441-4. 30804337 PMC6389936

[17] Wu Z, Li Y. Grifolin exhibits anti-cancer activity by inhibiting the development and invasion of gastric tumor cells. Oncotarget. 2017;8(13):21454–21460. doi:10.18632/oncotarget.15250. 28206955 PMC5400597

[18] Yan L, Liu Y, Ma XF, Hou D, Zhang Y-H, Sun Y, Shi S-S, Forouzanfar T, Lin H-Y, Fan J. et al. Triclabendazole induces pyroptosis by activating caspase-3 to cleave GSDME in breast cancer Cells. Front Pharmacol. 2021;12:670081. doi:10.3389/fphar.2021.670081. 34305590 PMC8297466

[19] Chen S, Zhang L, Li M, Zhang Y, Sun M, Wang L, Lin J, Cui Y, Chen Q, Jin C. et al. Fusobacterium nucleatum reduces METTL3-mediated m(6)A modification and contributes to colorectal cancer metastasis. Nat Commun. 2022;13(1):1248. doi:10.1038/s41467-022-28913-5. 35273176 PMC8913623

[20] Jin L, Chen Y, Cheng D, He Z, Shi X, Du B, Xi X, Gao Y, Guo Y. YAP inhibits autophagy and promotes progression of colorectal cancer via upregulating bcl-2 expression. Cell Death Disease. 2021;12(5):457. doi:10.1038/s41419-021-03722-8. 33963173 PMC8105309

[21] Han Y, Zhang X, Guan M, Huo C, Yu C, Hu B, Cai J. RASSF4 inhibits cell proliferation and increases drug sensitivity in colorectal cancer through YAP/Bcl-2 pathway. J Cell Mol Med. 2022;26(12):3538–3547. doi:10.1111/jcmm.17395. 35611809 PMC9189339

[22] Tao RH, Kobayashi M, Yang Y, Kleinerman ES. Exercise inhibits doxorubicin-induced damage to cardiac vessels and activation of Hippo/YAP-Mediated apoptosis. Cancers Basel. 2021;13(11):2740. doi:10.3390/cancers13112740. 34205942 PMC8198139

[23] Zitvogel L, Ayyoub M, Routy B, Kroemer G. Microbiome and Anticancer Immunosurveillance. Cell. 2016;165(2):276–287. doi:10.1016/j.cell.2016.03.001. 27058662

[24] Scott TA, Quintaneiro LM, Norvaisas P, Lui PP, Wilson MP, Leung K-Y, Herrera-Dominguez L, Sudiwala S, Pessia A, Clayton PT. et al. Host-microbe Co-metabolism dictates cancer drug efficacy in C. elegans. Cell. 2017;169(3):442–456.e18. doi:10.1016/j.cell.2017.03.040. 28431245 PMC5406385

[25] Zhang S, Yang Y, Weng W, Guo B, Cai G, Ma Y, Cai S. Fusobacterium nucleatum promotes chemoresistance to 5-fluorouracil by upregulation of BIRC3 expression in colorectal cancer. J Exp Clin Cancer Res. 2019;38(1):14. doi:10.1186/s13046-018-0985-y. 30630498 PMC6327560

[26] Liu Z, Li Y, Zhu Y, Li N, Li W, Shang C, Song G, Li S, Cong J, Li T. et al. Apoptin induces pyroptosis of colorectal cancer cells via the GSDME-dependent pathway. Int J Biol Sci. 2022;18(2):717–730. doi:10.7150/ijbs.64350. 35002520 PMC8741846

[27] Geng M, Wang L, Li P. Correlation between chemosensitivity to anticancer drugs and bcl-2 expression in gastric cancer. Int J Clin Exp Pathol. 2013;6:2554–2559. 24228120 PMC3816827

[28] Beale PJ, Rogers P, Boxall F, Sharp SY, Kelland LR. BCL-2 family protein expression and platinum drug resistance in ovarian carcinoma. Br J Cancer. 2000;82(2):436–440. doi:10.1054/bjoc.1999.0939. 10646901 PMC2363292

[29] Kim DW, Kim KO, Shin MJ, Ha JH, Seo SW, Yang J, Lee FY. siRNA-based targeting of antiapoptotic genes can reverse chemoresistance in P-glycoprotein expressing chondrosarcoma cells. Mol Cancer. 2009;8(1):28. doi:10.1186/1476-4598-8-28. 19445670 PMC2689171

[30] Yu FX, Zhao B, Guan KL. Hippo pathway in organ size control, tissue homeostasis, and cancer. Cell. 2015;163(4):811–828. doi:10.1016/j.cell.2015.10.044. 26544935 PMC4638384

[31] Sanchez-Vega F, Mina M, Armenia J, Chatila WK, Luna A, La KC, Dimitriadoy S, Liu DL, Kantheti HS, Saghafinia S. et al. Oncogenic signaling pathways in the cancer genome atlas. Cell. 2018;173(2):321–337.e10. doi:10.1016/j.cell.2018.03.035. 29625050 PMC6070353

[32] Lee KW, Lee SS, Kim SB, Sohn BH, Lee H-S, Jang H-J, Park Y-Y, Kopetz S, Kim SS, Oh SC. et al. Significant association of oncogene YAP1 with poor prognosis and cetuximab resistance in colorectal cancer patients. Clin Cancer Res. 2015;21(2):357–364. doi:10.1158/1078-0432.CCR-14-1374. 25388162 PMC4513664

[33] Hu L, Liu Y, Kong X, Wu R, Peng Q, Zhang Y, Zhou L, Duan L. Fusobacterium nucleatum facilitates M2 macrophage polarization and colorectal carcinoma progression by activating TLR4/NF-κB/S100A9 cascade. Front Immunol. 2021;12:658681. doi:10.3389/fimmu.2021.658681. 34093546 PMC8176789

[34] Chen T, Li Q, Wu J, Wu Y, Peng W, Li H, Wang J, Tang X, Peng Y, Fu X. et al. Fusobacterium nucleatum promotes M2 polarization of macrophages in the microenvironment of colorectal tumours via a TLR4-dependent mechanism. Cancer Immunol Immunother. 2018;67(10):1635–1646. doi:10.1007/s00262-018-2233-x. 30121899 PMC11028377

[35] Chen Y, Peng Y, Yu J, Chen T, Wu Y, Shi L, Li Q, Wu J, Fu X. Invasive Fusobacterium nucleatum activates beta-catenin signaling in colorectal cancer via a TLR4/P-PAK1 cascade. Oncotarget. 2017;8(19):31802–31814. doi:10.18632/oncotarget.15992. 28423670 PMC5458249

[36] Deng Y, Lu J, Li W, Wu A, Zhang X, Tong W, Ho KK, Qin L, Song H, Mak KK. et al. Reciprocal inhibition of YAP/TAZ and NF-κB regulates osteoarthritic cartilage degradation. Nat Commun. 2018;9(1):4564. doi:10.1038/s41467-018-07022-2. 30385786 PMC6212432

[37] Wang S, Xie F, Chu F, Zhang Z, Yang B, Dai T, Gao L, Wang L, Ling L, Jia J. et al. YAP antagonizes innate antiviral immunity and is targeted for lysosomal degradation through IKKɛ-mediated phosphorylation. Nat Immunol. 2017;18(7):733–743. doi:10.1038/ni.3744. 28481329

[38] Sato T, Stange DE, Ferrante M. et al. Long-term expansion of epithelial organoids from human colon, adenoma, adenocarcinoma, and Barrett’s epithelium. Gastroenterology. 2011;141(5):1762–1772. doi:10.1053/j.gastro.2011.07.050. 21889923

[39] Sato T, van Es JH, Snippert HJ, Stange DE, Vries RG, van den Born M, Barker N, Shroyer NF, van de Wetering M, Clevers H. et al. Paneth cells constitute the niche for Lgr5 stem cells in intestinal crypts. Nature. 2011;469(7330):415–418. doi:10.1038/nature09637. 21113151 PMC3547360

