# Supplementary figures and images for: Fusobacterium nucleatum induces chemoresistance in colorectal cancer by inhibiting pyroptosis via the Hippo pathway
Source: Gut Microbes. 2024 Mar 27;16(1):2333790. doi: 10.1080/19490976.2024.2333790 (PMC10978024; doi:10.1080/19490976.2024.2333790)

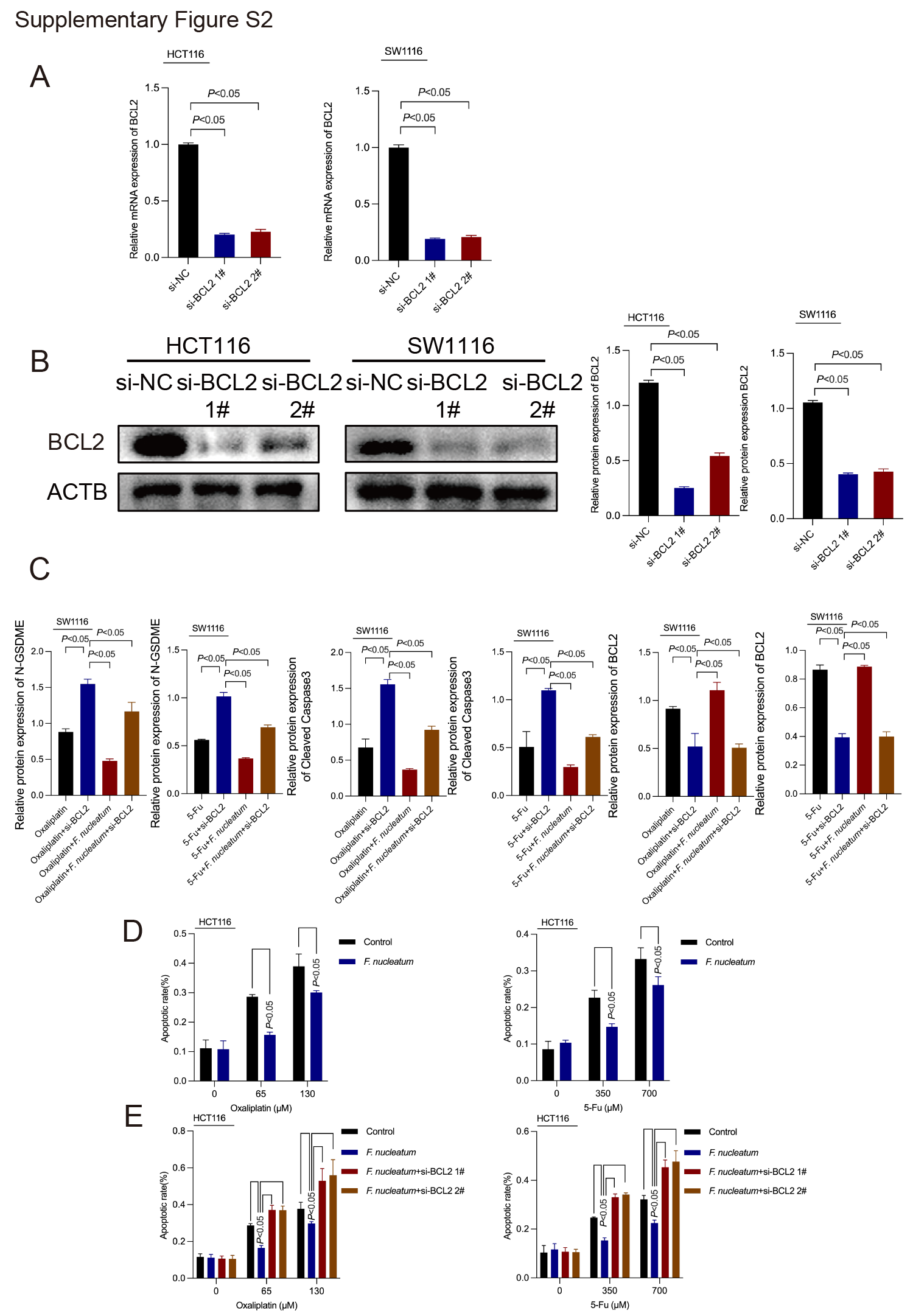

Supplement: Supplementary Figure S2.tif [file KGMI_A_2333790_SM1094.tif]

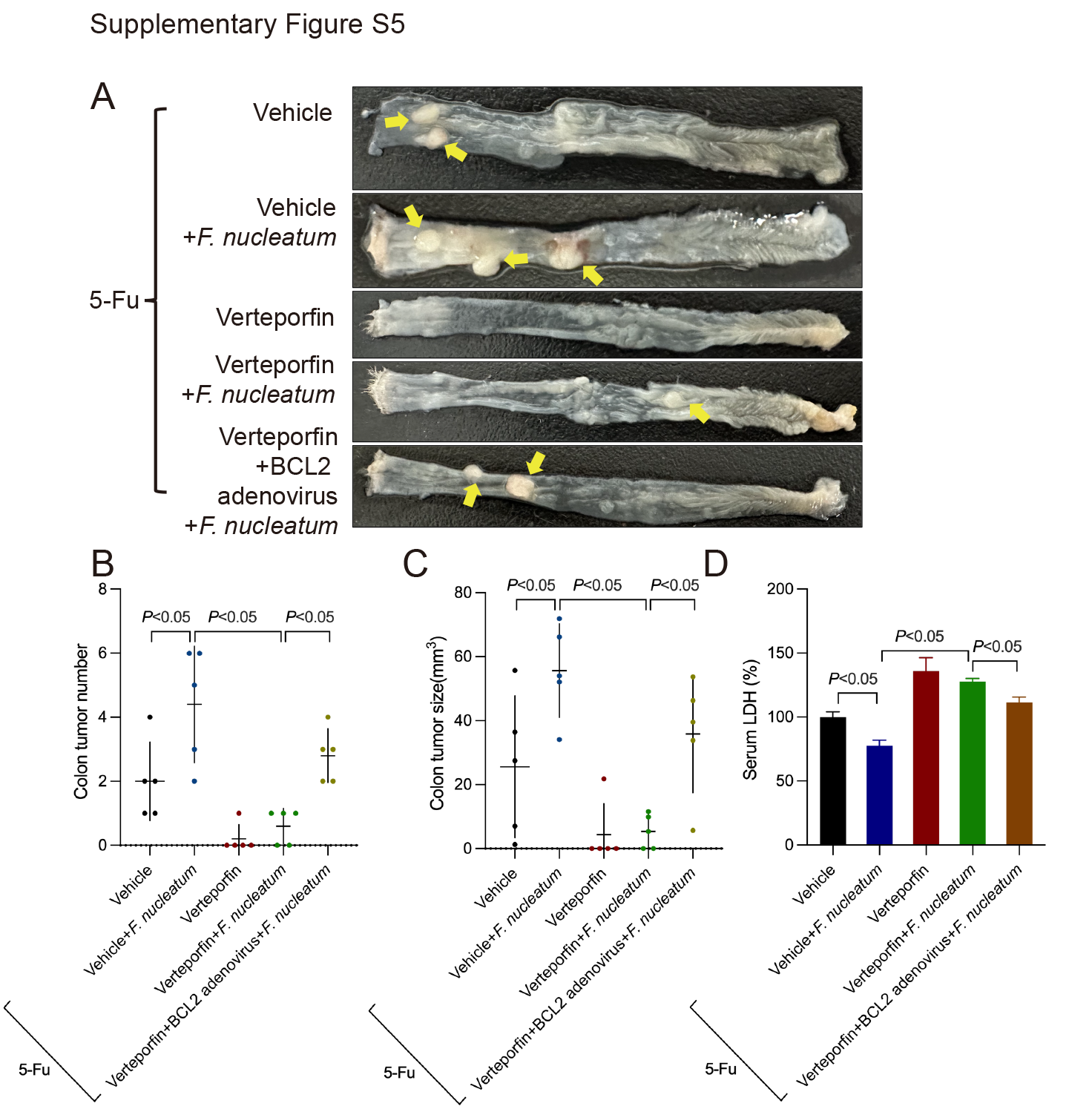

Supplement: Supplementary Figure S5.tif [file KGMI_A_2333790_SM1091.tif]

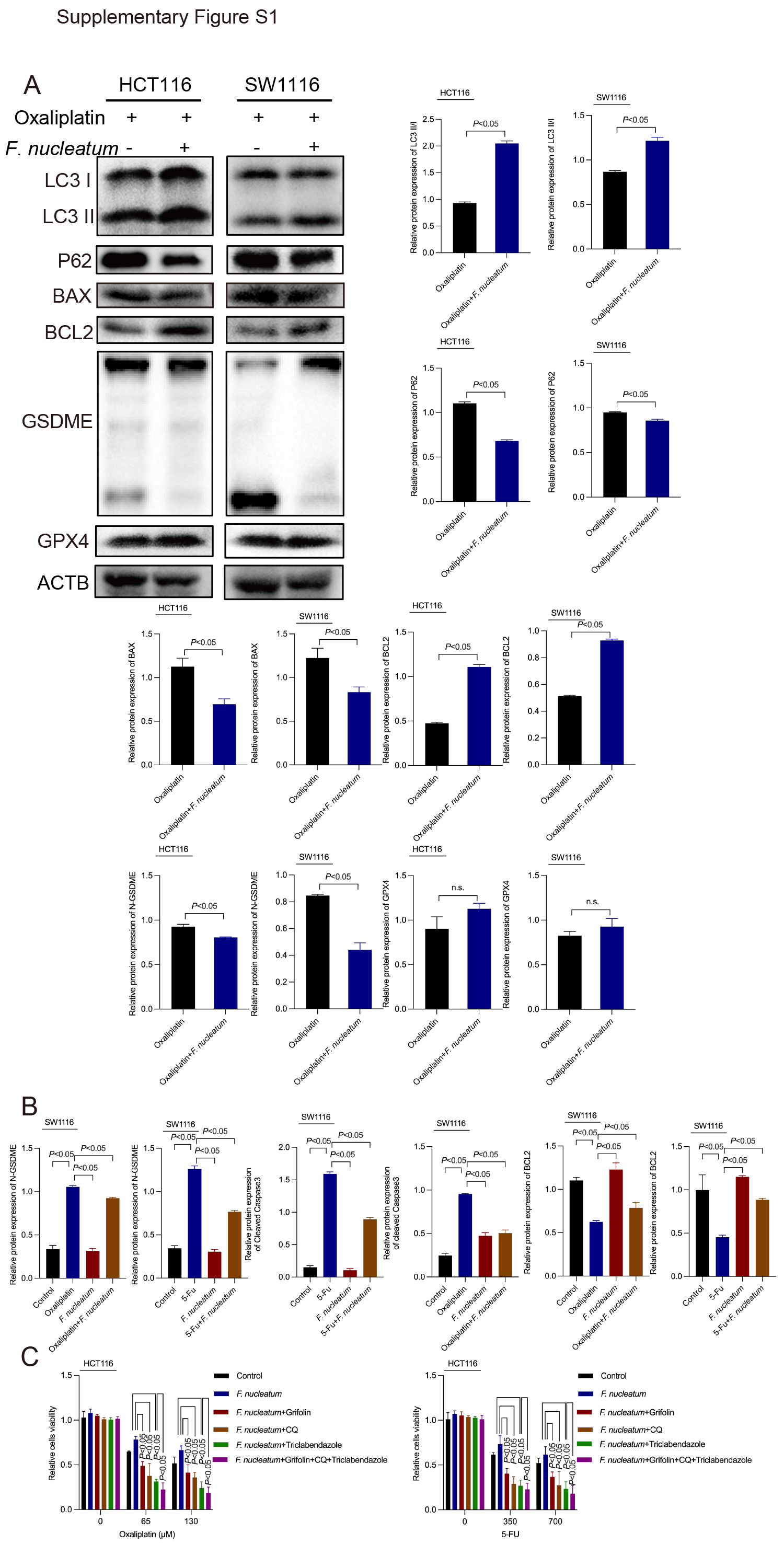

Supplement: Supplementary Figure S1.tif [file KGMI_A_2333790_SM1090.tif]

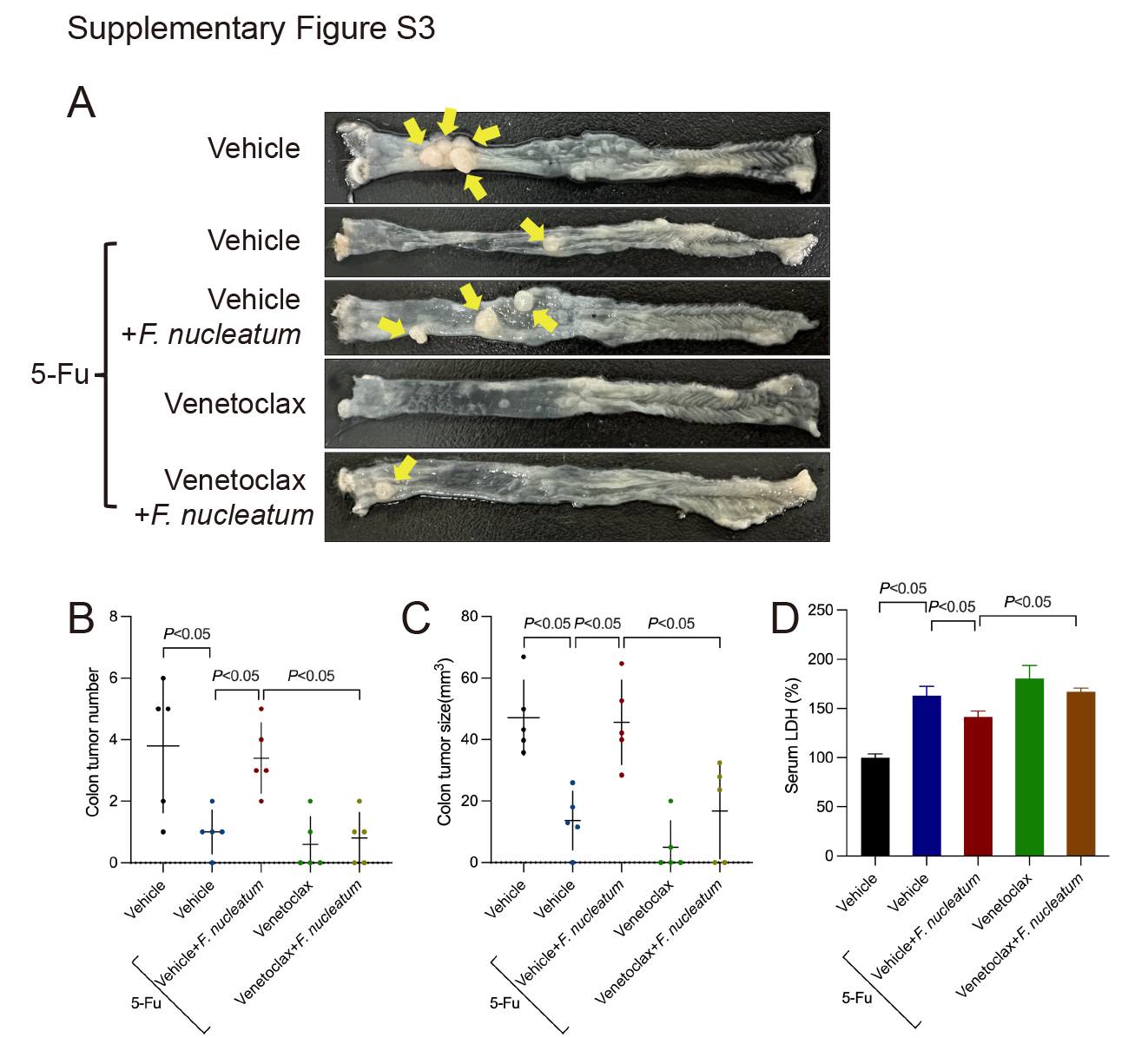

Supplement: Supplementary Figure S3.tif [file KGMI_A_2333790_SM1089.tif]

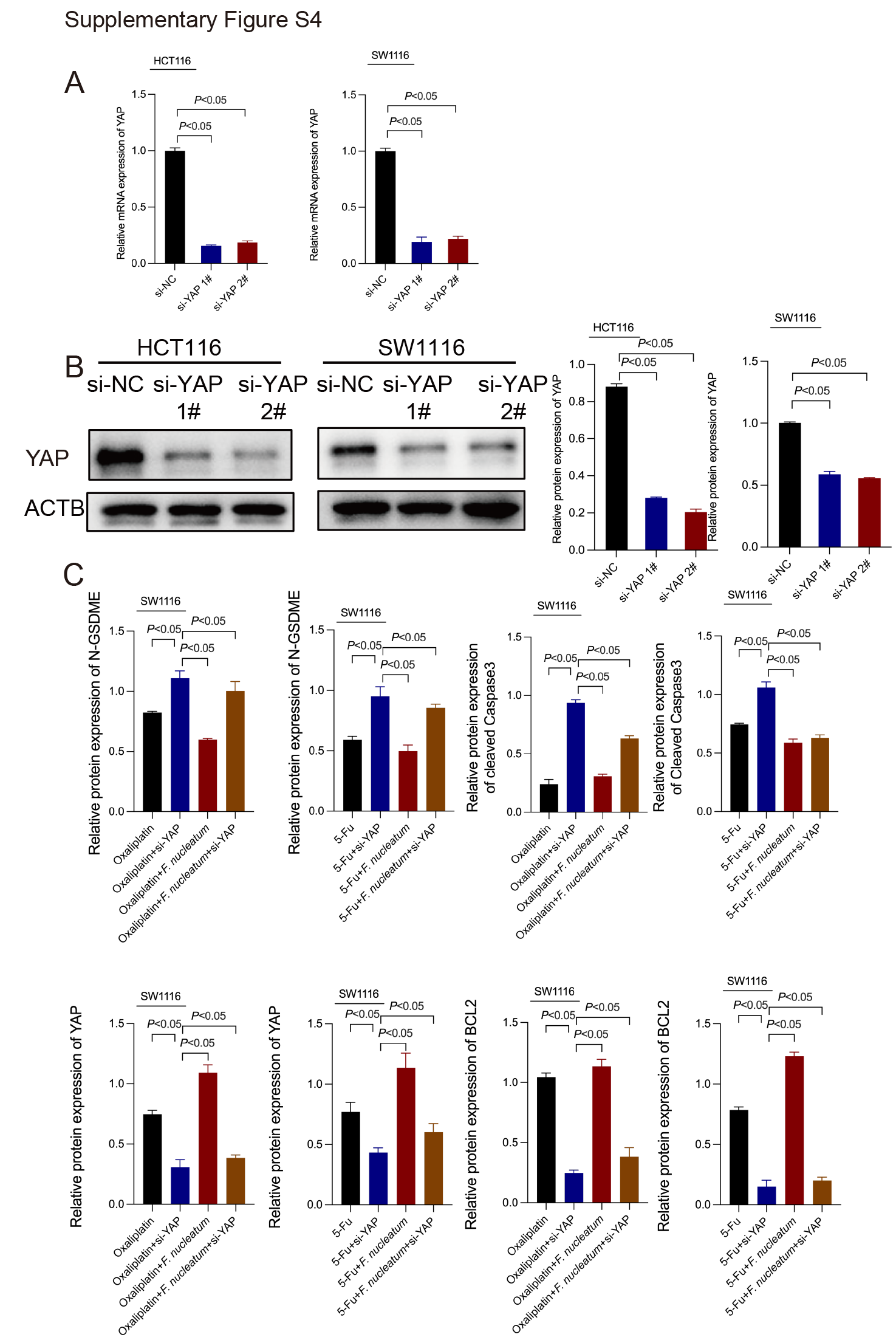

Supplement: Supplementary Figure S4.tif [file KGMI_A_2333790_SM1088.tif]

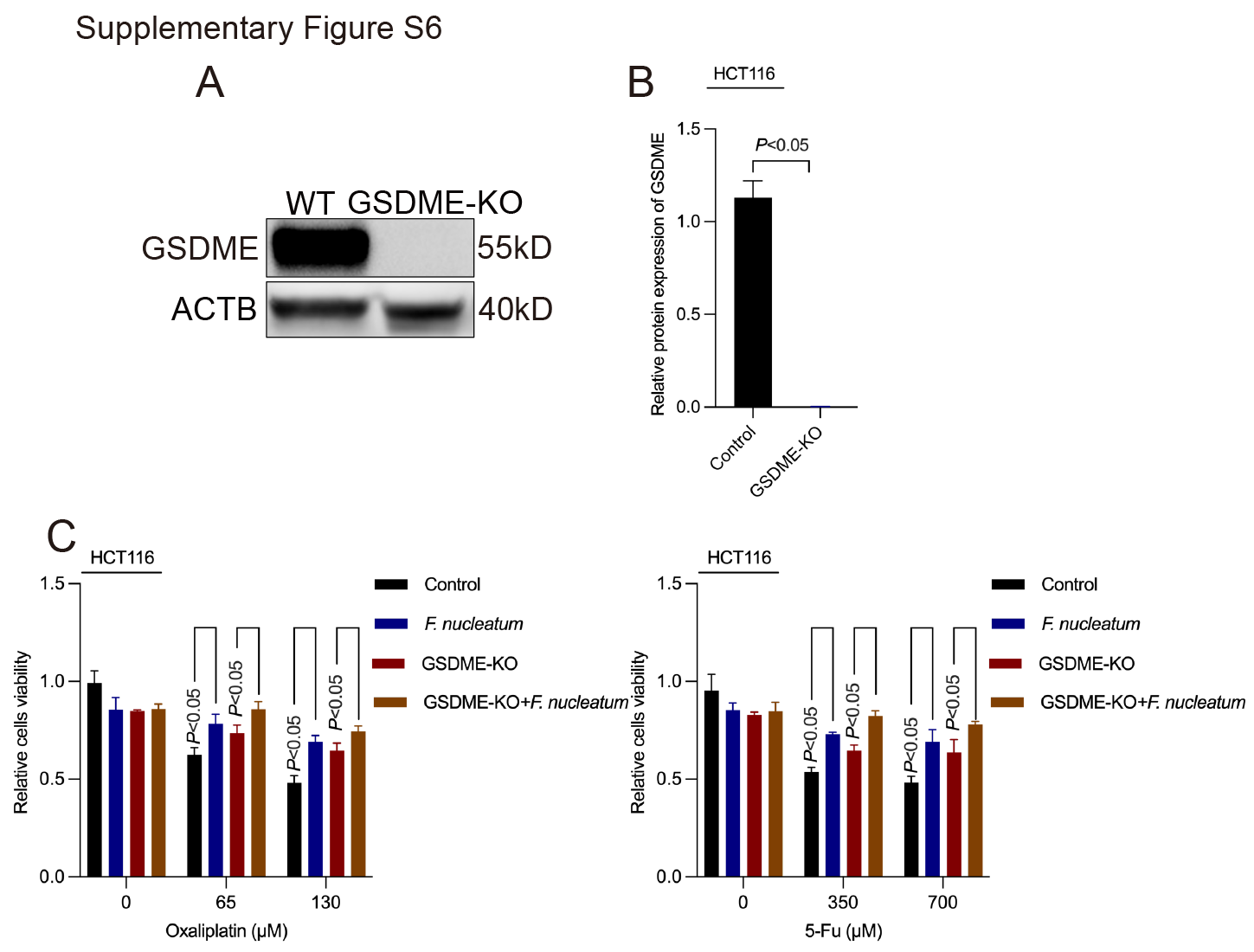

Supplement: Supplementary Figure S6.tif [file KGMI_A_2333790_SM1087.tif]
