# Supplementary material for: Fusobacterium nucleatum induces chemoresistance in colorectal cancer by inhibiting pyroptosis via the Hippo pathway
Source: Gut Microbes. 2024 Mar 27;16(1):2333790. doi: 10.1080/19490976.2024.2333790 (PMC10978024; doi:10.1080/19490976.2024.2333790)
Supplement: Supplementary Figure Legends clean.docx [file KGMI_A_2333790_SM1092.docx]

Figure S1. *F. nucleatum* inhibits chemotherapy-induced pyroptosis

(A)The effect of *F. nucleatum* intervention on autophagy, apoptosis, pyroptosis and ferroptosis in CRC cells under oxaliplatin treatment was measured by Western Blot. (B) Western Blot results quantification of the effect of *F. nucleatum* on the expression of pyroptosis-related proteins in chemotherapy-treated SW1116 cells. (C) Cell viability after treatment with chemotherapy, *F. nucleatum*, apoptosis agonists Grifolin, autophagy lysosomal inhibitor Chloroquine (CQ) and pyroptosis agonists Triclabendazolein in CRC cells was detected by CCK8 assay. All experiments were performed in biological triplicates.

Figure S2. Knockdown of BCL2 affects the inhibition of *F. nucleatum* on pyroptosis

(A and B) The mRNA and protein expression of BCL2 following treatment of CRC cells with siRNAs was detected by qRT-PCR and Western Blot. (C) Quantification of Western Blot results from knockdown of BCL2 reversed effects of *F. nucleatum* on pyroptosis-related proteins in SW1116 cells. (D) The apoptotic rates of CRC cells treated with *F. nucleatum* were measured by flow cytometry. *F. nucleatum* repressed the apoptosis of CRC cells induced by chemotherapy. (E) Downregulated BCL2 could rescue the modulation of *F. nucleatum* on apoptosis. All experiments were performed in biological triplicates.

Figure S3. *F. nucleatum* prompted chemotherapy resistance by regulating BCL2

(A) *Apc^Min/+^* mice were used to examine the effect of BCL2 inhibition on *F. nucleatum*-induced chemoresistance. (B and C) Tumor numbers (B) and size (C) were calculated after mouse sacrifice. (E) The release of serum LDH in *Apc^Min/+^* mice was measured. Bars indicate S.D.

Figure S4. *F. nucleatum* suppress chemotherapy-induced pyroptosis by regulating YAP

(A and B) The mRNA and protein expression of YAP following treatment of CRC cells with siRNAs was detected by qRT-PCR and Western Blot. (C) Quantification of Western Blot results of inhibition of YAP affecting the regulation of proteins involved in the pathway by *F. nucleatum* in SW1116 cells. All experiments were performed in biological triplicates.

Figure S5. *F. nucleatum* induced chemotherapy resistance by modulating YAP.

(A) The effect of YAP repression on *F. nucleatum*-induced chemoresistance was detected in *Apc^Min/+^* mice. (B and C) Tumor numbers (B) and size (C) were calculated after mouse sacrifice. (E) The release of serum LDH in *Apc^Min/+^* mice was detected. Bars indicate S.D.

Figure S6. GSDME-KO partially blocked *F. nucleatum*-induced chemotherapy resistance

(A and B) The protein expression of GSDME in stable GSDME-knockout cells obtained via CRISPR/Cas9 was detected by Western Blot. (C) Cell viability after treatment with chemotherapy and *F. nucleatum* in GSDME-knockout CRC cells was detected by CCK8 assay. All experiments were performed in biological triplicates.
